# A machine learning model for prediction of sarcopenia in patients with Parkinson’s Disease

**DOI:** 10.1371/journal.pone.0296282

**Published:** 2024-01-02

**Authors:** Minkyeong Kim, Doeon Kim, Heeyoung Kang, Seongjin Park, Shinjune Kim, Jun-Il Yoo

**Affiliations:** 1 Department of Neurology, Gyeongsang National University Hospital, Jinju, South Korea; 2 Department of Neurology, Gyeongsang National University College of Medicine, Jinju, South Korea; 3 Department of Data Analysis, Korea Expressway Corporation, Gimcheon, South Korea; 4 Department of Biomedical Research Institute, Inha University Hospital, Incheon, South Korea; 5 Department of Orthopedic Surgery, Inha University Hospital, Incheon, South Korea; Gyeongsang National University, REPUBLIC OF KOREA

## Abstract

**Objective:**

Patients with Parkinson’s disease (PD) have an increased risk of sarcopenia which is expected to negatively affect gait, leading to poor clinical outcomes including falls. In this study, we investigated the gait patterns of patients with PD with and without sarcopenia (sarcopenia and non-sarcopenia groups, respectively) using an app-derived program and explored if gait parameters could be utilized to predict sarcopenia based on machine learning.

**Methods:**

Clinical and sarcopenia profiles were collected from patients with PD at Hoehn and Yahr (HY) stage ≤ 2. Sarcopenia was defined based on the updated criteria of the Asian Working Group for Sarcopenia. The gait patterns of the patients with and without sarcopenia were recorded and analyzed using a smartphone application. The random forest model was applied to predict sarcopenia in patients with PD.

**Results:**

Data from 38 patients with PD were obtained, among which 9 (23.7%) were with sarcopenia. Clinical parameters were comparable between the sarcopenia and non-sarcopenia groups. Among various clinical and gait parameters, the average range of motion of the hip joint showed the highest association with sarcopenia. Based on the random forest algorithm, the combined difference in knee and ankle angles from standing still before walking to the maximum angle during walking (Kneeankle_diff), the difference between the angle when standing still before walking and the maximum angle during walking for the ankle (Ankle_dif), and the min angle of the hip joint (Hip_min) were the top three features that best predict sarcopenia. The accuracy of this model was 0.949.

**Conclusions:**

Using smartphone app and machine learning technique, our study revealed gait parameters that are associated with sarcopenia and that help predict sarcopenia in PD. Our study showed potential application of advanced technology in clinical research.

## Introduction

Sarcopenia is associated with aging, which is characterized by progressive skeletal muscle loss and related functional deterioration [1]. Sarcopenia is more prevalent in individuals with Parkinson’s disease than it is in the general population [2]. Furthermore, the prevalence of sarcopenia tends to increase as the disease progresses, which is associated with functional disabilities such as falls and dysphagia [3–5]. Several translational research has shown a shared mechanism between Parkinson’s Disease and sarcopenia such as inflammation, but it has not been fully elucidated to date [6]. Given that sarcopenia is associated with a poor clinical outcome in Parkinson’s Disease, and exercise and dietary intervention have shown beneficial effects, early diagnosis and intervention are warranted [7].

Patients with Parkinson’s Disease have characteristic gait patterns; a stooped posture, narrowed base, slow gait speed, and stride length variability [8]. As the disease progresses, freezing, festination, loss of postural reflex, and falling appear. These gait patterns not only help differentiate Parkinson’s Disease from healthy controls but also reflect clinical characteristics [9]. Patients with postural instability and gait difficulty (PIGD) phenotype demonstrate reduced velocity, stride length, and range of motion of pelvis and hip when compared to those with tremor dominant phenotype [10]. Gait velocity and step length correlate with medication status in patients suffering from motor fluctuation [11]. Furthermore, Parkinson’s Disease gait is easily affected by various factors such as dual tasks, visual or auditory cues, or neuromuscular dysfunction [12, 13].

In this study, we focused on the clinical significance of sarcopenia in Parkinson’s Disease, especially its impact on gait pattern. We analyzed the gait patterns of patients with early-stage Parkinson’s Disease with and without sarcopenia using an app-derived program and explored if gait parameters could be utilized to predict sarcopenia in Parkinson’s Disease based on machine learning.

## Methods

### Patients

This study was approved by the Institutional Review Board of Gyeongsang National University Hospital, Jinju, Korea (approval no. 2020-09-007 and 2022-04-027), and written informed consent were obtained from all patients. Patients were enrolled from December 2020 to May 2023 among those who visited the hospital. We enrolled patients with Parkinson’s Disease at Hoehn and Yahr (HY) stage ≤ 2 from the Movement Disorder Clinic of Gyeongsang National University Hospital, Jinju, Korea. The diagnosis of Parkinson’s Disease was based on the Movement Disorder Society (MDS) Clinical Diagnostic Criteria for Parkinson’s Disease [14]. Patients with Parkinson’s Disease suffering from other neurological diseases such as stroke or neuromuscular disorders, and having a history of spine surgery or spinal cord injury were excluded. Those who were accompanied by hyperthyroidism, cancer, tuberculosis, liver cirrhosis, a renal disease requiring hemodialysis, and obesity (body mass index BMI ≥ 30 kg/m^2^) that could lead to muscle wasting were also excluded.

### Procedure

Clinical information and sarcopenia profiles of patients with Parkinson’s Disease were collected as follows: sex; age at Parkinson’s Disease onset; disease duration; MDS-Unified Parkinson’s Disease Rating Scale (MDS-UPDRS) III and HY stage during “on” state [15, 16]; Tremor and PIGD scores, calculated by the sum of MDS-UPDRS items 2.10, 3.15, 3.16, 3.17, and 3.18, and the sum of MDS-UPDRS items 2.12, 2.13, 3.10, 3.11, and 3.12, respectively [17]; levodopa equivalent daily dose (LEDD); right and left asymmetry, present if right-left differences in the MDS-UPDRS items 3.3, 3.4, 3.6, 3.8, 3.15, 3.16, and 3.17b-e were ≥ 5 points [18].

To classify patients with sarcopenia among those with Parkinson’s disease, we applied the criteria set forth by the Asian Working Group for Sarcopenia (AWGS 2019). This entailed the utilization of the following standards: low appendicular skeletal muscle mass index (ASMI) for males (< 7.0 kg/m2) and females (< 5.7 kg/m2), coupled with low handgrip strength (< 28 kg for males, < 18 kg for females), or a low 6-meter walking velocity (< 1.0 m/s) [19]. The ASMI was calculated as the sum of skeletal muscle mass in the upper and lower extremities divided by the square of height, which was assessed by bioelectrical impedance analysis (BIA, InBody770, InBody Co., Ltd, Seoul Korea). Handgrip strength was measured by a Smedley-type dynamometer (Takei, Japan).

### Data collection

Participants were asked to walk a distance of 6-meters twice without any set velocity limit in order to acquire videos for pose estimation analysis. Due to the absence of a standardized protocol for recording angles, measurement methods and tools, a height of 90cm corresponding to the abdominal region was selected as the designated recording protocol. Subsequently, to ensure sufficient inclusion of patients’ movements within the video frame, cameras were positioned at 1-meter intervals along the central axis of the 6-meter linear path, both for lateral views and frontal perspectives. For frontal views, cameras were also situated at 1-meter intervals starting from the destination point. Recording was conducted using a smartphone (LM-V510N, LG Electronics Inc., Republic of Korea).

### Data analysis

The gait pattern was analyzed using an application called ’Deevo.gait,’ which was integrated into the Dr.Log App. The Deevo.gait application utilizes Google’s Blazepose model, known for its accurate estimation of joint positions within the body. With the Blazepose model, keypoints representing shoulder, elbow, wrist, hip, knee, and ankle joints on both sides are identified from the video after the body is detected. Videos in which the patient’s body couldn’t be reliably detected, such as those containing two or more individuals, were excluded from the study. The keypoint coordinates consist of 3D data encompassing x, y, and z values, derived from the fusion of coordinates captured by the two smartphone cameras. Finally, clinical-related variables and gait function-related variables generated from pose estimation coordinate data were selected, and detailed information about them is presented in S1 Data.

### Statistical analysis

When comparing the groups with and without sarcopenia, continuous clinical-related and physical function-related variables were expressed as ’mean ± standard deviation,’ while categorical variables were presented as ’number of patients (%).’ For the analysis of continuous variables between groups, the Shapiro-Wilk test was conducted to assess normality. In cases where variables met the assumption of normal distribution, Student’s t-test was employed; when normality assumptions were not met, the Mann-Whitney U test was utilized to determine the significance of inter-group differences.

To investigate correlations between variables, the Pearson’s chi-square test was applied when distribution assumptions were met. If not, Fisher’s exact test was used. A significance level of *P* < 0.05 was considered statistically significant.

Ultimately, a random forest model was employed to construct a predictive model for sarcopenia in patients with Parkinson’s Disease. The entire patient cohort was randomly divided into training (75.0%) and testing (25.0%) sets. In the process of dividing the data, allocation was randomized, and the training was iterated 10 times. Subsequently, the results for training and testing from these 10 iterations were aggregated to compute the confusion matrix, from which we calculated precision, recall, specificity, accuracy, and f1-score. Additionally, Gini importance, which calculates variable importance, was utilized to identify variables that discriminate sarcopenia using the testing set.

All figures were created and statistical analyses were conducted using R version 4.1.2 (R Core Team, R Foundation for Statistical Computing, Vienna, Austria, 2021). The ggplot2 package was used for visualizations, the random forest package for analysis, and the dplyr package for data preparation. Furthermore, the variables used in the random forest analysis and their corresponding descriptions are as presented in Table 1.

**Table 1:** Clinical and gait parameters used in sarcopenia prediction models.

Variables	Description
ASM	Appendicular Skeletal Muscle Mass
ASMI	Appendicular Skeletal Muscle Mass index
low_ASMI	Indicating ’yes’ if the Appendicular Skeletal Muscle Mass Index is low, with thresholds for males: < 7.0 kg/m^2^ and females: < 5.7 kg/m^2^
handgrip_strength	Measurement of handgrip strength
low_handgrip_strength	Indicating ’yes’ if handgrip strength is low, with thresholds for males: < 28 kg and females: < 18 kg
Walking_velocity	6-meter walking velocity
Low_6m_walk	Indicating ’yes’ if 6-meter walking velocity is < 1.0 m/s
Sex	Gender of the individual
disease_duration	The duration of the disease at the time of enrollment
age_enrolment	The age of the individual at the time of enrollment
BMI	A measure of body fat based on height and weight
Height	The height of the individual in centimeters
Weight	The weight of the individual in kilograms
PIGD_score	Sum of MDS-UPDRS 2.12, 2.13, 3.10, 3.11, and 3.12
Sarcopenia	Presence of Sarcopenia
shoulder_range	Shoulder angle range (max-min)
shoulder_min	Minimum shoulder angle
shoulder_max	Maximum shoulder angle
shoulder_mean	Average shoulder angle
hip_range	Hip angle range (max-min)
hip_min	Minimum hip angle
hip_max	Maximum hip angle
hip_mean	Average hip angle
knee_range	Knee angle range (max-min)
knee_min	Minimum knee angle
knee_max	Maximum knee angle
knee_mean	Average knee angle
ankle_range	Ankle angle range (max-min)
ankle_min	Minimum ankle angle
ankle_max	Maximum ankle angle
ankle_mean	Average ankle angle
hip_dif	Difference between the angle when standing still before walking and the maximum angle during walking (hip)
ankle_dif	Difference between the angle when standing still before walking and the maximum angle during walking (ankle)
knee_dif	Difference between the angle when standing still before walking and the maximum angle during walking (knee)
kneeankle_dif	Difference between the angle when standing still before walking and the maximum angle during walking (knee + ankle)
hipankle_dif	Difference between the angle when standing still before walking and the maximum angle during walking (hip + ankle)
hipknee_dif	Difference between the angle when standing still before walking and the maximum angle during walking (hip + knee)
all_max_dif	Difference between the angle when standing still before walking and the maximum angle during walking (hip + knee + ankle)

MDS-UPDRS, Movement Disorder Society-Unified Parkinson’s Disease Rating Scale; PIGD, postural instability and gait difficulty; BMI, Body Mass Index; ASMI, appendicular skeletal muscle mass index

## Results

### Clinical characteristics

Overall, 38 patients with Parkinson’s Disease were enrolled in this study, among which, 9 (23.7%) were with sarcopenia whereas 29 (76.3%) were without sarcopenia (sarcopenia versus non-sarcopenia group, respectively). Sarcopenia profiles, baseline demographic, and clinical information are presented in Table 2. Patients with Parkinson’s Disease and sarcopenia demonstrated higher frequencies of low ASMI and low handgrip strength whereas 6-meter walking velocity and clinical characteristics were comparable between the groups.

**Table 2:** Clinical characteristics of Parkinson’s Disease patients.

	PD with sarcopenia (*n* = 9)	PD without sarcopenia (*n* = 29)	*p*-value
ASMI (*n*, /m^2^)	9 (100%)	1 (3.4%)	< 0.001
	5.3 ± 0.3	6.9 ± 0.9	< 0.001
Handgrip strength (*n*, kgf)	7 (77.8%)	6 (20.7%)	0.002
	16.5 ± 3.6	26.1 ± 7.1	< 0.001
6 m walking (*n*, m/s)	7 (77.8%)	15 (51.7%)	0.167
	1.25 ± 1.74	0.93 ± 0.21	0.149
Sex (M:F)	1:8	11:18	0.223
Age of onset	65.1 ± 7.6	61.1 ± 8.1	0.201
Disease duration (*y*)	5.3 ± 3.3	5.9 ± 4.4	0.973
MDS-UPDRS III	28.3 ± 10.7	31.7 ± 9.3	0.366
HY stage	1.9 ± 0.3	1.9 ± 0.3	1.000
Tremor score	1.4 ± 2.6	2.2 ± 2.9	0.497
PIGD score	2.3 ± 1.3	2.5 ± 2.3	0.756
Asymmetry (*n*, %)	0 (0.0%)	4 (13.8%)	0.554
LEDD	417.5 ± 246.1	583.2 ± 726.4	0.945
Weight (kg)	49.2 ± 2.4	62.5 ± 7.6	< 0.001
Height (cm)	147.0 ± 3.8	159.3 ± 6.9	< 0.001
BMI (kg/m^2^)	22.8 ± 1.7	24.6 ± 2.5	0.055

PD, Parkinson’s disease; M, male; F, female; MDS-UPDRS, Movement Disorder Society-Unified Parkinson’s Disease Rating Scale; HY, Hoehn and Yahr; PIGD, postural instability and gait difficulty; LEDD, levodopa equivalent daily dose; ASMI, appendicular skeletal muscle mass index; MNA, Mini Nutritional Assessment; IPAQ, International Physical Activity Questionnaire

### Results of random forest

**Fig 1:**
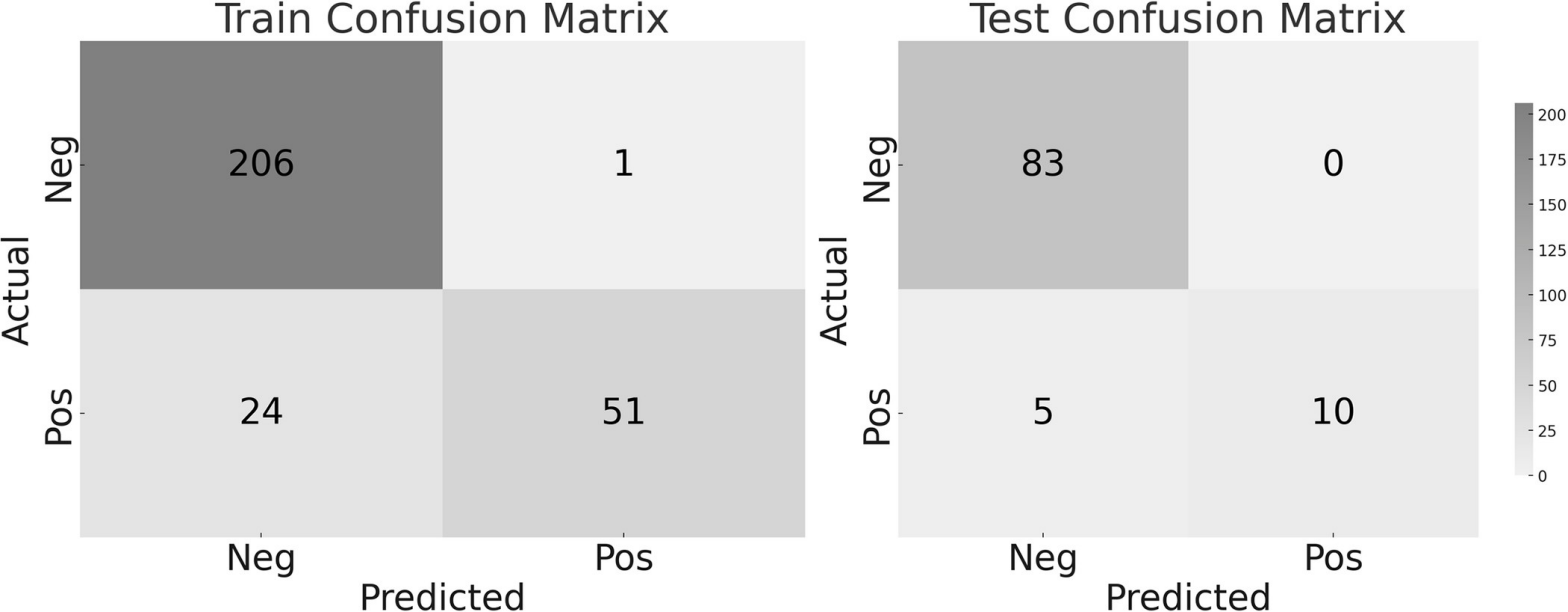
Confusion matrix of the random forest model used to predict sarcopenia (left side: train set, right side: test set).

After dividing the data into 75% for training and 25% for testing, the confusion matrix results from the random forest model, obtained after 10 iterations, are as displayed in Fig 1. Additionally, the performance metrics derived from this confusion matrix are presented in Table 3. The performance metrics delineated in Table 3 encapsulate the random forest model’s proficiency. For the training set, precision is exceptionally high at 0.981, indicating that the model accurately identifies the positive class, while recall at 0.680 suggests that some positive cases are missed. Specificity is nearly perfect at 0.995, demonstrating the model’s effectiveness in ruling out negative cases. The accuracy of the training set stands at 0.911, showing a high overall rate of correct classifications, and the F1-Score at 0.803 indicates a strong balance between precision and recall. In the test set, the model achieves perfect precision and specificity, signifying no false positives or negatives were identified. The recall is slightly lower at 0.667, pointing to the potential for missed positive cases. The accuracy climbs to 0.949, denoting a very high level of correct predictions across the board, and the F1-Score remains high at 0.800, reflecting the model’s consistent performance in predicting sarcopenia.

**Table 3:** Performance metrics of random forest model.

	Precision	Recall	Specificity	Accuracy	F1-Score
Train	0.981	0.680	0.995	0.911	0.803
Test	1.000	0.667	1.000	0.949	0.800

**Fig 2:**
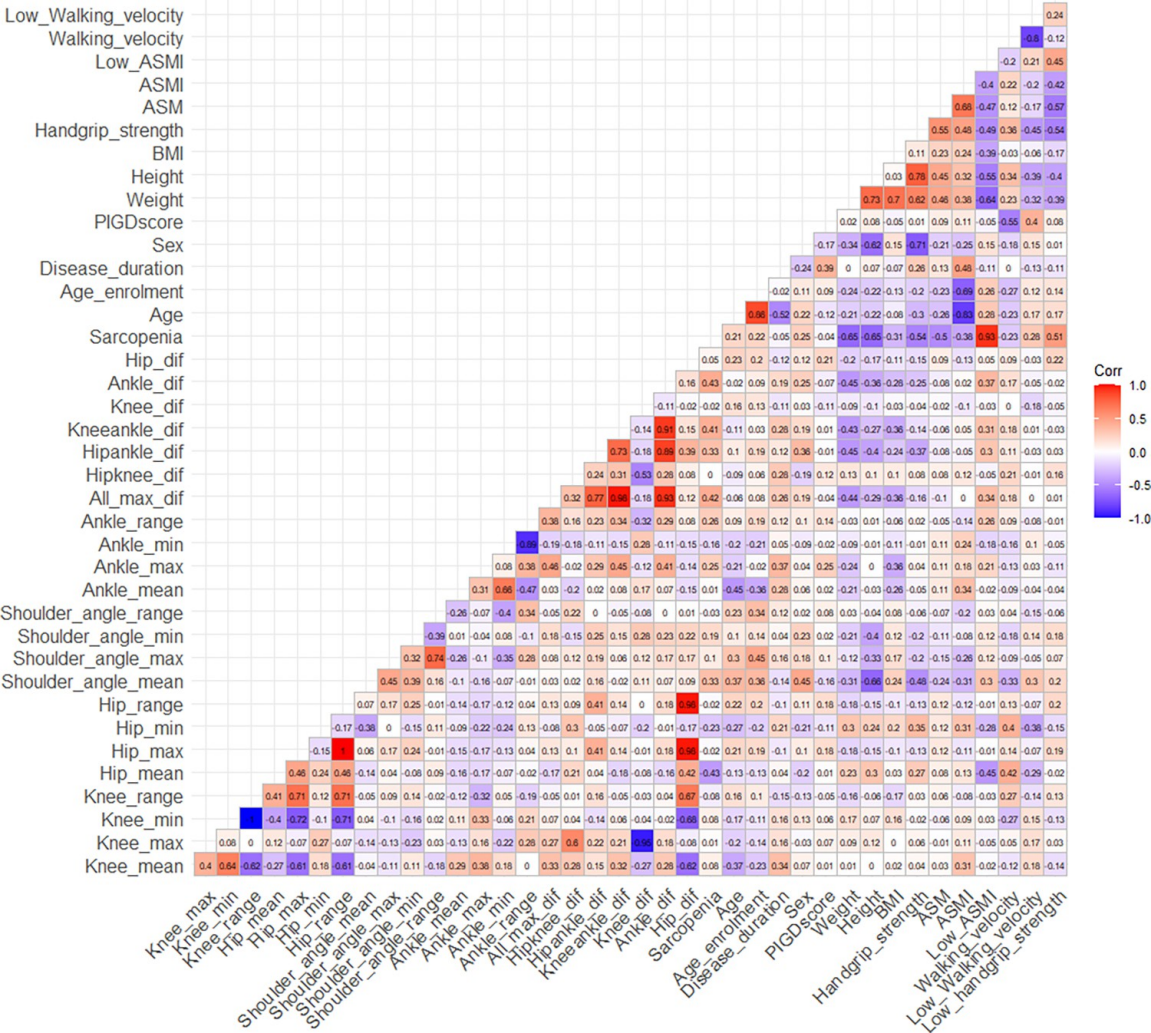
Correlation matrix between the absence of sarcopenia and other variables in Parkinson’s Disease patients.

Building upon the previous analysis, factors significantly correlated with sarcopenia in Parkinson’s Disease patients were identified. The average hip angle (hip_mean), a measure of the mean angle at the hip joint during movement, showed a notable negative correlation (*r* = -0.429), suggesting that a smaller mean hip angle is associated with sarcopenia. Similarly, the ankle_dif, which captures the change in ankle angle from a stationary position to its maximum during walking, had a positive correlation (*r* = 0.429). This indicates that greater changes in ankle movement correlate with the presence of sarcopenia. The all_max_dif, representing the overall maximum difference in joint angles during walking, had a correlation (*r* = 0.424) close to that of the ankle, signifying its relevance. Moreover, the kneeankle_dif, denoting the combined difference in knee and ankle angles from standing still to maximum movement, was also positively correlated (*r* = 0.407). Expanding on other variables, a low Appendicular Skeletal Muscle Mass Index (low_ASMI) showed a very strong positive correlation (*r* = 0.932), indicating that lower ASMI values are highly indicative of sarcopenia. This index is critical as it benchmarks muscle mass against height squared, providing thresholds for sarcopenia diagnosis in males and females. Weight and Height were inversely correlated with sarcopenia, with coefficients of -0.651 and -0.649, respectively, implying that lower body weight and stature are associated with higher sarcopenia prevalence. Handgrip strength, an assessment of muscle strength, was negatively correlated (*r* = -0.543) with sarcopenia, highlighting the condition’s impact on muscular function. Lastly, the absolute value of ASM displayed a negative correlation (*r* = -0.498), further underlining the relationship between muscle mass and sarcopenia. These findings are visually synthesized in Fig 2.

**Fig 3:**
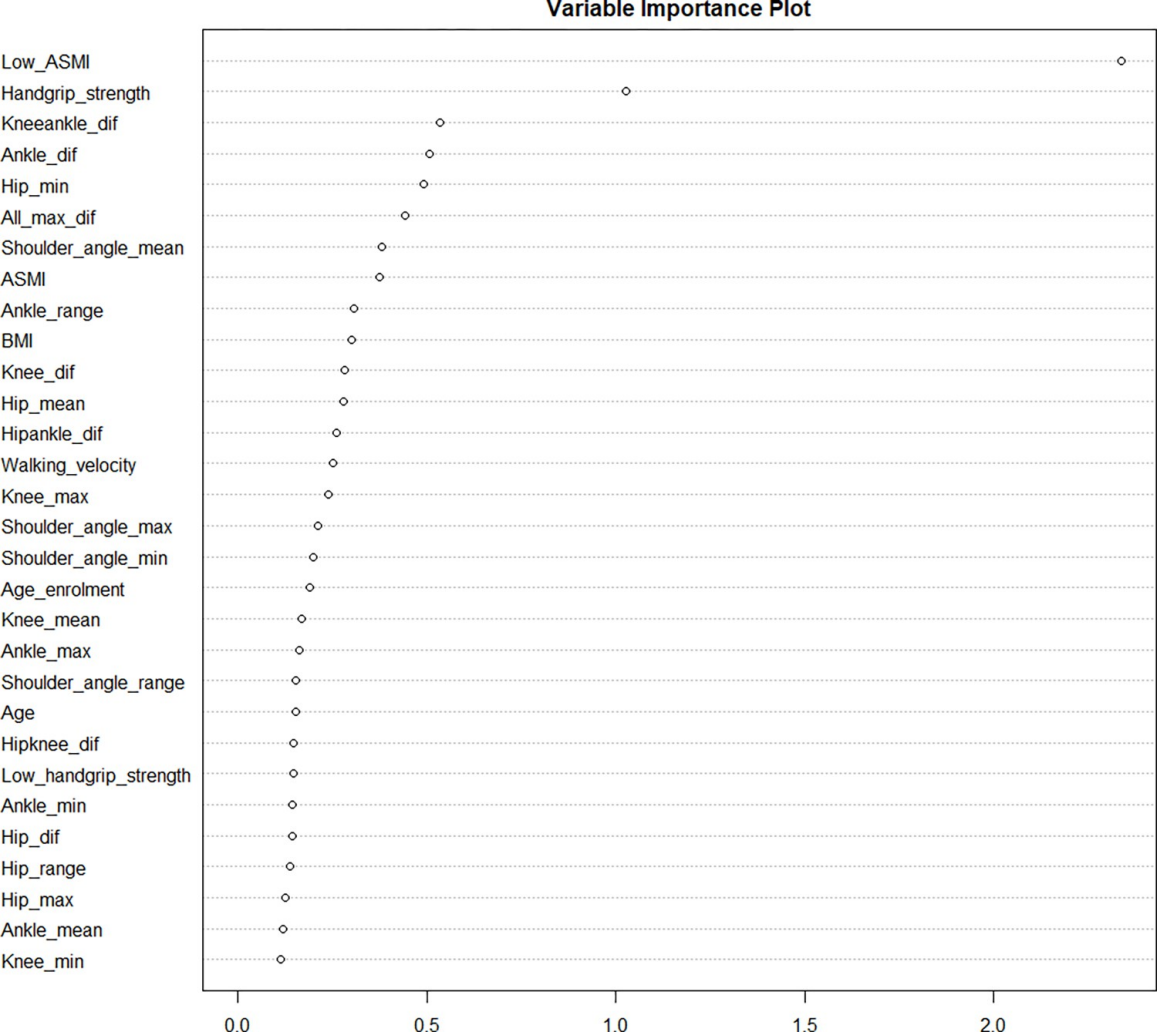
Variable importance plot in the random forest model.

The importance of variables for distinguishing sarcopenia in Parkinson’s Disease, as determined through the random forest analysis, was ranked with Low_ASMI and Handgrip_strength at the forefront, followed by Kneeankle_dif, Ankle_dif, Hip_min, among others. Excluding Low_ASMI and Handgrip_strength, which are directly related to sarcopenia criteria, the significance of the remaining variables suggests that the differences in ankle, hip, and knee angles from a standing to walking onset are of notable importance (Fig 3).

## Discussion

In this study, we assessed the gait patterns of patients with Parkinson’s Disease with and without sarcopenia using Dr.Log APP installed with Deevo.gait. Except for variables that attribute to the definition of sarcopenia, the average range of motion of the hip joint showed the strongest association with sarcopenia among various clinical and gait parameters. Based on the random forest algorithm, Kneeankle_dif, Ankle_dif, and Hip_min turned out to be the top three important features to predict sarcopenia among various clinical and gait parameters. The accuracy of this model was 0.949, achieving excellent prediction power.

To date, quantitative gait analysis has been performed using an in-hospital laboratory system that exhibits video recordings, motion capture cameras, or force plates [20]. Recent advancement in technology diversified gait analysis using smartphones or portable devices. In this study, we used a smartphone application to recognize locations of each participant’s joints while walking. As the application did not require markers attached to participants’ body parts or specialized motion capture cameras, it reduced space constraints and presented possible usability in non-hospital settings to analyze more natural gait patterns. Furthermore, we applied machine learning techniques to analyze gait pattern that had been utilized to differentiate Parkinson’s Disease gait from normal gait, detect freezing, or predict falls in Parkinson’s Disease in previous studies [21–23].

For obvious reasons, variables related to grip strength and ASM showed a strong association with sarcopenia. Except for variables that were used for defining sarcopenia, the movement of hip joint showed the strongest negative association with sarcopenia; that is, the smaller the range of motion of hip joint was, the higher probability of sarcopenia the patient had, or vice versa. This may be related with reduced step and stride length observed in both Parkinson’s Disease and sarcopenia. Furthermore, reduced range of motion of hip joint may make patients with Parkinson’s Disease and sarcopenia more susceptible to fall, fracture, delayed recovery, and mortality [24, 25].

Our study revealed that the relative difference between knee and ankle joints rather than the movement of the individual joints contributed the most to predicting sarcopenia in patients with Parkinson’s Disease. There is a coupling relationship between knee and ankle joints during flat path walking: during the swing phase, the ankle angle remains relatively constant as the knee angle changes, whereas the ankle angles changes at a constant rate as the knee angle increases during the stance phase [26]. Sarcopenia in patients with Parkinson’s Disease may have influenced this coupling relationship between knee and ankle joints, distinguishing the sarcopenia group from the non-sarcopenia group. This is in line with a previous study where healthy women with frailty demonstrated greater variability of knee and ankle joint angles than those without frailty [27].

It is noticeable that clinical characteristics did not play a significant role in predicting sarcopenia in Parkinson’s Disease (Table 2). Generally, sarcopenia was associated with the severity of Parkinson’s Disease motor symptoms and its incidence increased as the disease progressed [5]. However, MDS-UPDRS III, HY stage, and PIGD scores were comparable between sarcopenia and non-sarcopenia groups and were not as important as gait parameters in predicting sarcopenia based on our algorithm. As patients who could walk 6 meters by themselves (HY stage ≤ 2) were enrolled and were “on” medication state during the assessment, relatively similar motor scores between the two groups may have been obtained. This also explains that 6-meter walking velocity did not significantly (*p* = 0.167) differ between the two groups. As patients with Parkinson’s Disease often present with asymmetric motor symptoms, more or less affected side could be separately analyzed. However, the average range of motion of bilateral limb joints was used as a variable in the current study because only 10.5% of the enrolled patients exhibited clinical asymmetry and all of them were in the non-sarcopenia group [18].

This study has several limitations. First, the sample size was small. However, clinical information was collected with a standardized protocol. Furthermore, three-dimensional data expressed in x, y, and z coordinates was large enough to perform a random forest because it contained an average of 600-time series information per person. Second, although patients were requested to walk a fixed distance on an even path, each patient’s walking trajectory was not standardized. If treadmill or walkway rug had been utilized, we could have provided more controlled environment for walking. Third, we only used loci of each joint in three-dimensional space but if spatiotemporal gait parameters were combined, better prediction power could be obtained [28]. Lastly, sarcopenia diagnosis may change depending on diagnostic methods. We used BIA, hand grip strength, and 6-meter walking velocity to define sarcopenia, but its prevalence varied depending on the test methods such as dual-energy x-ray absorptiometry, short physical performance battery, or time up and go [29].

Patients with Parkinson’s Disease demonstrate characteristic gait patterns in nature, and various digital tools that calculate joint angles and ranges are actively applied in this field. As sarcopenia is associated with poor clinical outcome in Parkinson’s Disease, early diagnosis and intervention is important. We investigated the gait patterns of patients with Parkinson’s Disease and sarcopenia using an app-derived program and identified gait parameters that best predict sarcopenia in Parkinson’s Disease. Our model not only demonstrated excellent accuracy but also revealed that kinematic gait analysis using a smartphone application has clinical implications for predicting sarcopenia in early-stage patients with Parkinson’s Disease. Furthermore, we believe that our result would enable clinicians to gather gait data from daily lives and provide timely education on exercise or dietary modification for patients with Parkinson’s Disease. Further study in a large population is warranted.

## Supporting information

S1 Data (CSV)
